# Detection and identification of antimicrobial-resistant *Salmonella* in raw beef at Wolaita Sodo municipal abattoir, Southern Ethiopia

**DOI:** 10.1186/s41043-017-0131-z

**Published:** 2017-12-16

**Authors:** Wondimu Wabeto, Yishak Abraham, Antehun Alemayehu Anjulo

**Affiliations:** 1 Dubbo Preparatory School, Wolaita Zone, Ethiopia; 2 Department of Medical Laboratory, College of Health Sciences and Medicine, Wolaita Sodo University, P.O. Box: 138, Wolaita Sodo, Ethiopia

**Keywords:** Antimicrobial susceptibility pattern, Beef, Abattoir, Isolation, Identification, *Salmonella* spp.

## Abstract

**Background:**

The consumption of multidrug resistant *Salmonella* isolates along with a raw meat dish is directly relevant to the global public health crisis of antimicrobial resistance. All countries around the globe are suffering from food-borne diseases. In developing countries, more than one billion individuals suffering from gastroenteritis and around five million infected individuals die annually.

**Methods:**

A cross-sectional study was carried out from December 2015 to May 2016 to show the risk of *Salmonella* associated with consuming traditional raw meat dishes and to characterize the antimicrobial resistance profile at Wolaita Sodo municipal abattoir. Animals were being processed as part of the normal work of the abattoir, and 448 carcass samples were taken after getting a written consent from the municipality. Samples were transported to Wolaita Sodo University Microbiology Laboratory in an ice box within an hour of collection. Swab samples were pre-enriched in tetrathionate broth and Rappaport-Vassiliadis R10 broth. Broth culture was sub-cultured on xylose lysine deoxycholate and brilliant green agar and incubated at a temperature of 37 °C for overnight. Antimicrobial susceptibility test was done by disk diffusion method. Microbiological and observational data entry and analysis were done using Microsoft Excel 2007.

**Results:**

From the total 448 sampled carcasses, *Salmonella* growth was observed in 56 (12.5%) samples. The isolates had various resistance profiles, with resistance to 1 to 12 antimicrobial drugs. Tetracycline- and nitrofurantion-resistant isolates were frequent, 83.93 and 73.21% respectively, and followed by streptomycin-resistant isolates (66%). Ciprofloxacin-resistant isolates were rare (7%).

**Conclusion:**

*Salmonella* species contamination frequency was high in raw beef, and most of the isolates exhibited resistance to commonly used antibiotics. People living in the town and consuming the raw meat are at risk for developing diseases, and attention should be given to select antimicrobials in treating *Salmonella* infections in both animals and human being based on antimicrobial susceptibility test. Moreover, intersectoral working and developing one health approach is essential. Health information should be given to individuals who have the habit of eating raw meat. Training on sanitary and hygiene practice should be given to the abattoir workers.

**Electronic supplementary material:**

The online version of this article (10.1186/s41043-017-0131-z) contains supplementary material, which is available to authorized users.

## Background

The consumption of multidrug-resistant *Salmonella* isolates along with a raw meat dish is directly relevant to the global public health crisis of antimicrobial resistance. Food-borne diseases caused by non-typhoid *salmonella* still remains as a public health challenge. All countries around the globe are suffering from food-borne disease outbreaks. The problem is more serious in developing countries; more than one billion cases of gastroenteritis and five million individuals die annually [1].

Poor food handling and sanitation practices, inadequate food safety laws, weak regulatory systems, lack of financial resources, and awareness about proper food handling create a conducive environment for the spread of food-borne and food poisoning etiologic agents in Ethiopia [2]. According to Minister of Health (MoH) report, 60% of the disease burden in Ethiopia was related to poor hygiene and sanitation [3]. Factors that contribute for the emerging of food-borne disease outbreak were unsafe sources, contaminated raw food items, improper food storage, and poor personal hygiene during food preparation, inadequate cooling and reheating of food items, and a prolonged time lapse between preparing and consuming food items [4].

*Salmonella* spp. are associated with a variety of food item and known for the cause of gastroenteritis, enteric fever, and septicemia. Foods of animal and vegetable origin such as beef, poultry, pork, egg, raw dairy products, vegetables, fruits, and juices are susceptible to *Salmonella* colonization during production and storage.

Microorganisms contaminate meat in abattoir during slaughtering and spread from the exterior part of animals and from the intestinal tract. Moreover, they are added from knives, cloths, air, workers, carts, boxes, and equipments. These microorganisms begin to multiply and spoil the meat if the environment is suitable for their growth. Food handlers in a carrier state or having an acute infection play a significant role in transmitting infection [5–7].

Studies showed that contaminations of raw meat with *Salmonella* may occur by contaminated feeds, during transportation of animals to abattoir, slaughtering operation and fecal contamination of edible organs, and storage, distribution, and preparation for consumption. Contamination of equipment, utensils, and personal hygiene of food handlers help to spread *Salmonella* [8–13].

The occurrence and distribution of *Salmonella* in Ethiopia was high. In recent years, the number of outbreaks of *Salmonella* in humans has been increasing from time to time. Much more was known about the extent of food-borne illness and its severity. Meanwhile, the effort has not been made to overcome the problem. Thus, food-borne pathogen continued to be a public health challenge in Ethiopia. Studies indicated that high percentages of *Salmonella typhi* isolates have been found to be resistant for antimicrobial agents [14].

In Ethiopia, eating raw meat is an indication of wealth. There is a popular traditional dish known locally as “KITFO” which is prepared from minced beef and, most of the time, it is consumed raw or moderately cooked. The habit of raw meat consumption and the presence of *Salmonella* in minced beef create a conducive environment to develop infection in the community [15].

Global trends in antimicrobial use in food animals in 2010 at 63,151 tons. It projects that antimicrobial consumption will rise by 67% by 2030 and nearly double in Brazil, Russia, India, China, and South Africa [16].

A study conducted in Bishoftu, Ethiopia, demonstrated the problems in clinical practice of veterinary medicine like problems in generic prescribing, incorrect diagnosis, and non-availability of standard veterinary treatment guideline and drug formulary [17].

The emerging of multiple drug-resistant *Salmonella* to commonly used antimicrobials has become a threat in both public health and veterinary sectors in Ethiopia [18]. The extensive use of the first-line drugs has led to the development of multiple drug resistance (MDR) at a level which could pose a serious problem in the near future. MDR is defined as resistance of an isolate to three or more antimicrobial drugs tested [19]. People living in the southern part of Ethiopia regularly eat raw meat, but nothing is known about the prevalence of *Salmonella* in raw beef. Therefore, this study intended to show the risk of *Salmonella* associated with consuming traditional raw meat dishes and to characterize the antimicrobial resistance profile at Wolaita Sodo municipal abattoir.

## Methods

### Study area

This study was conducted in Wolaita Sodo town administration, which is located in the southern part of Ethiopia and lies between 1600 and 2100 m above sea level, 330 km away from Addis Ababa, the capital city of Ethiopia. The total population range and household data of the town is 110,659 and 14,551, respectively, in 2015 (information from the study area finance office). The rainfall is bimodal and characterized by medium rainy season from February to May and high rainy season from July to September. The dry season extends from October to January. There are three sub-cities and one municipality. However, there is only one abattoir. More than 25 cattles are slaughtered per day in the abattoir, and majority of people have the habit of eating raw meat in the study area. It is considered as an indication of wealth.

### Study design

A cross-sectional study was carried out from December 2015 to May 2016.

### Sample size determination

The sample size was determined by taking a 17.7% prevalence; it was taken from a study entitled “Isolation, identification, antimicrobial susceptibility test and public awareness of *Salmonella* on raw goat meat at Dire Dawa Municipal Abattoir, eastern Ethiopia” [20], assuming a 5% margin of error and a 95% confidence level and a design effect of 2 and 10% for non-response rate. The calculated sample size was 448.

### Sampling technique, sample collection, and procedures

A total of 448 cattle were included in this study. Two carcass samples were taken from each cattle according to the guideline [21]. Then, the cattle carcasses were taken consecutively still to complete the final sample size.

The abdomen (flank), thorax (lateral), crutch, and breast (lateral) were the areas from the body parts of cattle. The sampling areas were delineated by using a 10 × 10 cm aluminum foil templates. A sterile cotton-tipped swab (2 × 3 cm) fit with shaft was first soaked in an approximately 10 ml of buffered peptone water (BPW) and rubbed over the delineated area horizontally and then vertically several times. Upon completion of the rubbing process, the swab was placed into the BPW used to wet the swab, breaking off the wooden shaft pressing against the inside of the universal bottle and disposed leaving the cotton swab in the universal bottle. Other swabs of the same types were used on the other marked areas and placed into the same container. A second dry sterile cotton swab of the same type was used as before over the entire sampled area as above and this swab placed into the same container. Finally, the samples were transported to Wolaita Sodo University Microbiology Laboratory in an ice box within an hour of collection. In addition to this, an observational checklist was used to assess carcass slaughtering and handling and to evaluate hygienic practices.

### Laboratory procedures

The swab samples were pre-enriched in an appropriate amount of BPW in a 1:9 ratio and were incubated at 37 °C for 24 h. Rappaport-Vassiliadis (RV) medium broth and Müller-Kauffman tetrathionate with novobiocin (MKTTn) broth were used for selective enrichment of the samples. About 0.1 ml of the pre-enriched samples was transferred into a tube containing 10 ml of RV broth and incubated at 42 °C for 24 h. Another 1 ml of the pre-enriched broth was transferred into a tube containing 10 ml of MKTTn broth and incubated at 37 °C for 24 h.

#### Plating out and identification

Xylose lysine desoxycholate (XLD) agar and brilliant green agar (BGA) plates were used for plating out and identification. A loop full of inoculums from each RV and MKTTn broth cultures were plated onto XLD and BGA plates and incubated at 37 °C for 24 h. After incubation, the plates were examined for the presence of typical and suspected colonies. Typical colonies of *Salmonella* [22], H_2_S-negative variants, and lactose-positive *Salmonella* were grown on XLD agar. Five typical or suspected colonies were selected from the selective plating media, were streaked onto the surface of pre-dried nutrient agar plates, and were incubated at 37 °C for 24 h. Biochemical tests were done according to ISO-6579, 2002. Triple sugar iron (TSI) agar, Simmon’s citrate indole motility (SIM) test, and citrate and urease tests were done.

#### Antimicrobial susceptibility tests

Antibiogram was done on Mueller-Hinton agar [23]. The following 12 antimicrobial agents’ resistance profiles, gentamicin (GEN 10 μg), tetracycline (TE 30 μg), amoxicillin/clavulanic acid (30 μg), ampicillin (AMP 10 μg), kanamycin (K-30 μg), nitrofuran (F 50 μg), streptomycin (S10 μg), ceftriaxone (CRO 30 μg), chloramphenicol (CHL 30 μg), clindamycin (DA 30 μg), nalidixic acid (NA 30 μg), and ciprofloxacin (CIP 5 μg), were done. All the antibiotic disks were purchased from Abtek Biological Ltd. (Liverpool). Inhibition diameters were interpreted according to the European Committee on Antimicrobial Susceptibility Instructions. The susceptibility, intermediate, and resistance characterizations were based on the recommendations from the CLSI and according to the manufacturer’s leaflet attached to the disks [24].

#### Quality control

*Pseudomonas aeruginosa* (ATCC-27853), *Staphylococcus aureus* (ATCC-25923), and *Escherichia coli* (ATCC-25922) were used as a quality control throughout the study for culture and antimicrobial susceptibility testing. All the strains were obtained from the Ethiopian Public Health Institute.

### Data entry and analysis

Microbiological and observational data entry and analysis were done using the Microsoft Excel 2007.

## Results

**Fig. 1:**
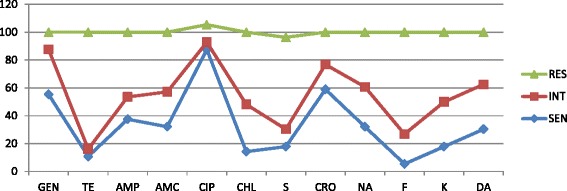
Multidrug resistant profile of *Salmonella* species isolated from raw beef samples at Wolaita Sodo municipal abattoir, 2016. Key: DA clindamycin, AMP ampicillin, AMC amoxicillin-clavulanic acid, GEN gentamicin, K kanamycin, CIP ciprofloxacin, CHL chloramphenicol, NA nalidixic acid, CRO ceftriaxone, F nitrofurantoin, S streptomycin, TE tetracycline

From the total 448 cattle carcasses, *Salmonella* species were detected in 56 (12.5%). Of these *Salmonella* species-contaminated samples, tetracycline-resistant isolates were detected in 47 samples (83.93%) followed by nitrofurantoin-resistant isolates, which was detected in 41 samples (73.21%). Streptomycin- and chloramphenicol-resistant isolates were detected in 37 (66.1%) and 29 (51.8%) samples respectively. Moreover, all isolated *Salmonella* spp. exhibited resistance to three or more antimicrobial agents and ciprofloxacin-susceptible isolates were detected in 49 samples (87.5%). Moreover, ceftriaxone- and gentamicin-susceptible isolates were detected in 33 and 31 samples, which were 58.91 and 55.4% respectively (Table 1) (Fig. 1) (Additional file 1).

**Table 1:** Antimicrobial resistance of *Salmonella* spp. isolated from raw beef samples at Wolaita Sodo municipal abattoir, 2016

Antimicrobial drugs	*Salmonella* spp. isolates		
	S (%)	I (%)	R (%)
GEN	31 (55.4%)	18 (32.1%)	7 (12.5%)
TE	6 (10.7%)	3 (5.4%)	47 (83.9%)
AMP	21 (37.5%)	9 (2.6%)	26 (46.4%)
AMC	18 (32.1%)	14 (25%)	24 (42.9%)
CIP	49 (87.5%)	3 (5.4%)	4 (7.1%)
CHL	8 (14.3%)	19 (33.9%)	29 (51.8%)
F	3 (5.4%)	12 (21.4%)	41 (73.2%)
S	10 (17.9%)	9 (2.6%)	37 (66.1%)
K	10 (17.9%))	17 (30.4%)	29 (51.8%))
CRO	33 (58.91%)	10 (17.9%)	13 (23.2%)
NA	18 (32.1%)	16 (28.6%)	22 (39.3%)
DA	17 (30.4%)	18 (32.1%)	21 (37.5%)
Total	224 (33.3%)	148 (22.0%)	300 (44.6%)

*GEN* gentamicin, *TE* tetracycline, *AMP* ampicillin, *AMC* amoxicillin-clavulanic acid, *CIP* ciprofloxacin, *CHL* chloramphenicol, *F* nitrofurantoin, *S* streptomycin, *K* kanamycin, *CRO* ceftriaxone, *NA* nalidixic acid, *DA* clindamycin

## Discussion

In this study, the contamination frequency of *Salmonella* species in raw beef was high (12.5%). The finding of this study was moderately higher than that of studies conducted in Modjo (8.3%) and Bishoftu (7.5%) [25, 26]. This difference could be due to the differences in the hygienic and sanitary practices in the abattoirs, the environment upon which the meat was slaughtered, and the water used in the processing of the meat. Wolaita Sodo municipal abattoir provides service for people residing in the town. Therefore, less emphasis was given to overcome the spread of potential pathogenic microorganisms when we compared with the referenced area. The abattoirs found in Modjo and Bishoftu town were export abattoirs. However, this study was in line with the studies conducted in Tigray region of Ethiopia; the *Salmonella* spp. prevalence was 16.4% [27] and at Dire Dawa abattoir 17.7% [20].

Studies conducted in different parts of Ethiopia showed that the prevalence of raw beef with *Salmonella* spp. varies from 5.3 to 17.7%, for instance, studies conducted in Addis Ababa, Bahir Dar, and Harar [28–31]. Though this study finding falls in the abovementioned range, there was a visible variation of *Salmonella* spp. prevalence. This might be because of the diversity in sampling methods, sampling seasons, and sampling as well as culturing techniques [32]. In addition, sanitation within the slaughterhouse and the cross-contamination of carcasses by contact with intestinal tracts during slaughter or processing were also important factors [33, 34].

With regard to the emerging of multidrug-resistant *Salmonella*, the following studies had been witnessing antimicrobial resistance of *Salmonella* to commonly used antimicrobials in both public health and veterinary sectors were increasing from time to time [35–42]. According to the present study, all 56 (100%) isolates were multidrug-resistant. This was similar with other studies conducted in Ethiopia, for instance, studies conducted in Jimma [35, 43] and Gondar [39, 41] and a study conducted among Ethiopian children. These studies had shown that *Salmonella* exhibited 100% resistance to multidrug. Furthermore, studies carried out around the globe [44–48] supported that *Salmonella* isolated from food of animal sources exhibited resistance to multidrug. The following studies reported the magnitude of multidrug-resistance of *Salmonella* isolates respectively as follows: 16%, 50% (from raw meats), 52%, 74.1 and 63.7% (from different types of samples), and 71.7 and 87.82%. This difference could be antimicrobial-resistant *Salmonella* were increasing due to the use of antimicrobial agents in food animals at the sub-therapeutic level or prophylactic doses, which might promote on-farm selection of antimicrobial-resistant strains and markedly increase the human health risks associated with consumption of contaminated meat products [36, 49, 50].

*Salmonella* isolates identified from food items and workers from Addis Ababa were resistant to the commonly used antibiotics including streptomycin, ampicillin, and tetracycline [50]. Furthermore, resistance of *Salmonella* isolates to the commonly used antimicrobials including ampicillin, streptomycin, nitrofurantoin, kanamycin and tetracycline were 100, 66.7, 58.3, and 33.3% respectively [51]. This was in line with the previous studies conducted to assess ampicillin-resistant *Salmonella* in South India 100% [52], Nigeria over 90% [53], and Cameroon 100% [54]. This study also indicated resistance of *Salmonella* isolates to commonly used antimicrobials including tetracycline, nitrofurantoin, streptomycin, kanamycin and ampicillin were 83.9, 73.2, 66, 50, and 46.43% respectively. A higher resistance rate was observed in this study when compared with the previous reports with the exception of ampicillin. In addition to this, resistance to amoxicillin-clavulanic acid and nalidixic acid were 42.9 and 39.3% respectively. This difference could be due to the increasing rate of inappropriate utilization of antibiotics, which favors selection pressure that increase the advantage of maintaining resistant genes in bacteria [55, 56]. Resistance to amoxicillin-clavulanic acid was observed, but it was introduced in the market in the last few years. The continuing development of antibiotic resistance could pose pressure to both animals and human being. Once the amoxicillin-clavulanic acid-resistant strains spread to human being, it might be difficult to treat with drug currently available in the market [57]. In addition to public health problems, it could lead to economic loss in the country due to loss of exporting meat and animal products.

Ciprofloxacin showed a good antimicrobial activity against the *Salmonella* isolates. From the 56 isolates, 49 (87.5%) were susceptible to ciprofloxacin which was comparable to the previous studies conducted in Ethiopia [49], Nigeria [53], and Addis Ababa [51]. The effectiveness of ciprofloxacin might be a recent introduction of the drug. It is not widely used in countries like Ethiopia and other African countries [51].

## Conclusions

*Salmonella* species contamination frequency was high in raw beef, and most of the isolates exhibited resistance to commonly used antibiotics. Tetracycline-resistant and ciprofloxacin-susceptible *Salmonella* species were frequently detected in raw beef. People living in the town and consuming the raw meat are at risk for developing diseases, and attention should be given to select antimicrobials in treating *Salmonella* infections in both animals and human being based on antimicrobial susceptibility test. Hence, intersectoral working and developing one health approach is essential. Health information should be given to individuals who have the habit of eating raw meat. Training on sanitary and hygiene practice should be given to the abattoir workers. WHO basic hygiene principles, which cover food safety procedures from the farm of origin to ante-mortem and post-mortem inspection to handling until the food is consumed, should be practiced.

## Additional file

Additional file 1: Table S1. Conventional biochemical test results of the samples that yielded growth on agar at Wolaita Sodo municipal abattoir, 2016. **Table S2** Antimicrobial susceptibility pattern of 56 isolates of *Salmonella* spp. at Wolaita Sodo municipal abattoir, 2016. **Figure S1** Antimicrobial susceptibility pattern of salmonella isolate at Wolaita Sodo Municipal Abattoir, 2016. (DOCX 37 kb)

## Abbreviations

BGA: Brilliant green agar
BPW: Buffered peptone water
MDR: Multiple drug resistance
MKTTn: Müller-Kauffman tetrathionate with novobiocin
MoH: Minister of Health
RV: Rappaport-Vassiliadis medium
SIM: Simmon’s citrate indole motility
TSI: Triple sugar iron
XLD: Xylose lysine desoxycholate
