# Supplementary material for: Detection and identification of antimicrobial-resistant Salmonella in raw beef at Wolaita Sodo municipal abattoir, Southern Ethiopia
Source: J Health Popul Nutr. 2017 Dec 16;36:52. doi: 10.1186/s41043-017-0131-z (PMC5732392; doi:10.1186/s41043-017-0131-z)
Supplement: Additional file 1: Table S1. — Conventional biochemical test results of the samples that yielded growth on agar at Wolaita Sodo municipal abattoir, 2016. Table S2 Antimicrobial susceptibility pattern of 56 isolates of Salmonella spp. at Wolaita Sodo municipal abattoir, 2016. Figure S1 Antimicrobial susceptibility pattern of salmonella isolate at Wolaita Sodo Municipal Abattoir, 2016. (DOCX 37 kb) [file 41043_2017_131_MOESM1_ESM.docx]

**Additional file 1**

Table S1: conventional biochemical test results of the samples that yielded growth on agar

| **Sample ID** | **TSI test** | | | | | | | | **Urease test** | **Citrate test** | **Indole** | **Motility** |
| --- | --- | --- | --- | --- | --- | --- | --- | --- | --- | --- | --- | --- |
|  | **Butt** | | **Slant** | **Gas** | | **H_2_S** | | |  |  |  |  |
| C04 | Yellow | Red | | | + | | | + | - | + | - | + |
| C10 | Yellow | Red | | | + | | | + | - | + | - | + |
| C19 | Yellow | Red | | | + | | | + | - | + | - | + |
| C20 | Yellow | Red | | | + | | | + | - | + | - | + |
| C23 | Yellow | Red | | | + | | | + | - | + | - | + |
| C31 | Yellow | Red | | | + | | | + | - | + | - | + |
| C34 | Yellow | Red | | | + | | | + | - | + | - | + |
| C36 | Yellow | Yellow | | | + | | | - | - | - | - | + |
| C41 | Yellow | Red | | | - | | | - | - | + | - | + |
| C47 | Yellow | Red | | | + | | | + | - | + | - | + |
| C55 | Yellow | Pink | | | + | | | + | - | + | - | + |
| C65 | Yellow | Red | | | + | | | + | - | + | - | + |
| C72 | Yellow | Pink | | | + | | | + | - | + | - | + |
| C78 | Yellow | Pink | | | + | | | - | - | + | - | + |
| C83 | Yellow | Red | | | - | | | - | - | + | - | + |
| C91 | Yellow | Red | | | - | | | + | - | + | - | + |
| C96 | Yellow | Red | | | + | | | + | - | + | - | + |
| C103 | Yellow | Pink | | | + | | | + | - | + | - | + |
| C112 | Yellow | Red | | | + | | | + | - | + | - | + |
| C117 | Yellow | Red | | | + | | | + | - | + | - | + |
| C118 | Yellow | Red | | | + | | | + | - | + | - | + |
| C121 | Yellow | Red | | | + | | | + | - | + | - | + |
| C127 | Yellow | Red | | | + | | | + | - | + | - | + |
| C134 | Yellow | Red | | | + | | | + | - | + | - | + |
| C152 | Yellow | Red | | | + | | | + | - | + | - | + |
| C158 | Yellow | Red | | | + | | | + | - | + | - | + |
| C160 | Yellow | Red | | | + | | | + | - | + | - | + |
| C164 | Yellow | Pink | | | + | | | + | - | + | - | + |
| C171 | Yellow | Red | | | - | | + | | - | + | - | + |
| C194 | Yellow | Red | | | + | | - | | - | + | - | + |
| C195 | Yellow | Red | | | + | | + | | - | + | - | + |
| C212 | Yellow | Pink | | | + | | - | | - | + | - | + |
| C225 | Yellow | Red | | | + | | + | | - | + | - | + |
| C230 | Yellow | Red | | | + | | + | | - | + | - | + |
| C234 | Yellow | Red | | | + | | + | | - | + | - | + |
| C252 | Yellow | Red | | | + | | + | | - | + | - | + |
| C254 | Yellow | Red | | | + | | + | | - | + | - | + |
| C255 | Yellow | Pink | | | - | | - | | - | + | - | + |
| C279 | Yellow | Yellow | | | + | | + | | - | + | - | + |
| C280 | Yellow | Red | | | + | | + | | - | + | - | + |
| C298 | Yellow | Red | | | + | | + | | - | + | - | + |
| C300 | Yellow | Red | | | + | | + | | - | + | - | + |
| C311 | Yellow | Red | | | + | | + | | - | + | - | + |
| C320 | Yellow | Red | | | + | | + | | - | + | - | + |
| C326 | Yellow | Red | | | + | | + | | - | + | - | + |
| C331 | Yellow | Red | | | + | | + | | - | + | - | + |
| C349 | Yellow | Red | | | + | | + | | - | + | - | + |
| C350 | Yellow | Red | | | + | | + | | - | + | - | + |
| C357 | Yellow | Red | | | + | | + | | - | + | - | + |

**Key**: + = positive – = negative

Table S2: Antimicrobial susceptibility pattern of 56 isolates of *Salmonella* spp

| **Isolates of *Salmonella* Spp.** | **Antimicrobial Susceptibility Pattern** | | | | | | | | | | | | **Total** | | |
| --- | --- | --- | --- | --- | --- | --- | --- | --- | --- | --- | --- | --- | --- | --- | --- |
|  | **GEN** | **TE** | **AMP** | **AMC** | **CIP** | **CHL** | **F** | **S** | **K** | **CRO** | **NA** | **DA** | **S (%)** | **I (%)** | **R (%)** |
| **1** | S | R | S | R | S | R | R | S | S | I | R | R | 5 (41%) | 1 (8.3%) | 6 (50%) |
| **2** | I | R | S | R | S | I | R | S | S | S | R | S | 6(50%) | 2(16.6%) | 4(33%) |
| **3** | R | R | R | R | S | R | R | I | S | I | R | R | 2(16.6%) | 2(16.6%) | 8(66.6%) |
| **4** | I | R | R | I | S | I | I | S | S | S | S | R | 5(41%) | 4(33%) | 3(25%) |
| **5** | R | R | S | I | S | S | I | R | S | S | I | R | 5(41%) | 3(25%) | 4(33%) |
| **6** | S | R | I | I | S | I | R | R | R | I | S | R | 3(25%) | 4(33%) | 5(41%) |
| **7** | I | R | S | S | S | S | S | R | R | S | I | R | 6(50%) | 2(16.6%) | 4(33%) |
| **8** | S | R | I | I | I | I | I | R | R | I | S | R | 2(16.6%) | 6(50%) | 4(33%) |
| **9** | I | R | S | I | S | I | R | R | S | S | I | R | 4(33%) | 4(33%) | 4(33%) |
| **10** | I | R | S | S | S | R | R | R | R | I | S | S | 5(41%) | 2(16.6%) | 5(41%) |
| **11** | I | R | I | I | S | S | R | R | S | S | I | R | 4(33%) | 4(33%) | 4(33%) |
| **12** | S | R | S | I | S | I | R | R | R | S | S | I | 5(41%) | 3(25%) | 4(33%) |
| **13** | S | R | I | S | S | R | R | R | S | R | I | R | 4(33%) | 2(16.6%) | 6(50%) |
| **14** | S | R | R | I | S | S | R | R | R | S | S | I | 5(41%) | 2(16.6%) | 5(41%) |
| **15** | S | R | R | I | S | R | R | R | I | I | R | R | 2(16.6%) | 3(25%) | 7(58%) |
| **16** | S | R | S | S | S | S | R | R | R | S | R | I | 6(50%) | 1(8.3%) | 5(41%) |
| **17** | S | R | R | I | S | R | R | S | I | I | S | R | 4(33%) | 3(25%) | 5(41%) |
| **18** | R | R | R | I | S | R | I | R | R | S | R | R | 2(16.6%) | 2(16.6%) | 8(66.6%) |
| **19** | S | R | R | S | S | R | I | R | I | I | R | S | 4(33%) | 3(25%) | 5(41%) |
| **20** | S | R | S | I | S | S | S | R | R | S | R | R | 6(50%) | 1(8.3%) | 5(41%) |
| **21** | S | S | R | R | I | R | R | R | I | R | I | I | 2(16.6%) | 4(33%) | 6(50%) |
| **22** | S | R | R | R | S | R | S | R | R | S | S | R | 5(41%) | - | 7(58%) |
| **23** | S | I | R | R | S | R | R | S | I | S | R | S | 5(41%) | 2(16.6%) | 5(41%) |
| **24** | S | R | R | R | S | R | I | R | R | S | S | R | 4(41%) | 1(8.3%) | 7(58%) |
| **25** | I | I | R | R | S | R | R | S | I | R | I | I | 2(16.6%) | 5(41%) | 5(41%) |
| **26** | S | R | R | R | S | R | I | I | R | S | S | R | 4(33%) | 2(16.6%) | 6(50%) |
| **27** | S | I | R | R | S | R | R | S | I | S | R | S | 5(41%) | 2(16.6%) | 5(41%) |
| **28** | S | R | I | S | S | R | I | R | R | S | S | R | 5(41%) | 2(16.6%) | 5(41%) |
| **29** | I | S | S | R | S | R | R | S | I | R | I | S | 5(41%) | 3(25%) | 4(33%) |
| **30** | S | S | S | S | S | R | I | R | R | S | S | R | 7(66.6%) | 1(8.3%) | 4(33%) |
| **31** | S | R | I | R | S | R | R | S | I | S | R | S | 5(41%) | 2(16.6%) | 5(41%) |
| **32** | I | R | S | S | S | R | I | R | R | S | I | R | 4(33%) | 3(25%) | 5(41%) |
| **33** | S | R | R | S | S | R | R | R | I | R | R | I | 3(25%) | 2(16.6%) | 7(58%) |
| **34** | S | R | R | I | S | R | I | R | R | R | R | S | 3(25%) | 2(16.6%) | 7(58%) |
| **35** | S | R | R | S | S | R | R | S | I | S | R | R | 5(41%) | 1(8.3%) | 6(50%) |
| **36** | S | R | I | S | S | R | I | I | R | R | R | I | 3(25%) | 4(33%) | 5(41%) |
| **37** | S | R | S | R | S | R | R | I | I | S | R | S | 5(41%) | 2(16.6%) | 5(41%) |
| **38** | I | S | R | S | S | R | R | I | R | R | I | I | 3(25%) | 4(33%) | 5(41%) |
| **39** | S | R | R | I | I | R | R | R | S | S | R | S | 4(33%) | 2(16.6%) | 6(50%) |
| **40** | S | R | S | S | S | R | R | I | R | S | R | I | 4(33%) | 2(16.6%) | 5(41%) |
| **41** | I | R | S | R | S | R | R | I | I | R | R | S | 3(25%) | 3(25%) | 6(50%) |
| **42** | I | R | R | S | S | I | R | R | R | R | I | I | 2(16.6%) | 4(33%) | 6(50%) |
| **43** | I | R | R | R | S | I | R | R | I | S | R | I | 2(16.6%) | 4(33%) | 6(50%) |
| **44** | R | R | S | R | S | I | R | R | R | S | R | I | 3(25%) | 2(16.6%) | 7(58%) |
| **45** | I | R | R | R | S | I | R | R | I | R | S | S | 3(25%) | 3(25%) | 6(50%) |
| **46** | R | R | I | R | S | I | R | R | R | S | I | I | 2(16.6%) | 4(33%) | 6(50%) |
| **47** | I | R | S | R | S | I | R | R | R | R | S | S | 4(33%) | 2(16.6%) | 6(50%) |
| **48** | I | R | R | R | S | I | R | R | R | S | I | I | 2(16.6%) | 4(33%) | 6(50%) |
| **49** | R | R | S | S | S | S | R | R | I | R | R | S | 5(41%) | 1(8.3%) | 6(50%) |
| **50** | I | R | I | R | S | I | R | I | R | S | S | I | 3(25%) | 5(41%) | 4(33%) |
| **51** | I | R | S | S | S | I | R | R | S | I | I | S | 5(41%) | 4(33%) | 3(25%) |
| **52** | R | R | R | R | S | I | R | I | R | S | S | I | 3(25%) | 3(25%) | 6(50%) |
| **53** | S | R | R | R | R | S | R | R | I | S | I | S | 4(33%) | 2(16.6%) | 6(50%) |
| **54** | S | R | S | S | R | I | R | R | R | I | I | I | 3(25%) | 4(33%) | 5(41%) |
| **55** | S | S | S | R | R | I | R | R | R | S | S | S | 6(50%) | 1(8.3%) | 5(41%) |
| **56** | S | S | R | S | R | I | R | R | R | S | S | I | 5(41%) | 2(16.6%) | 5(41%) |
| **Total** | | | | | | | | | | | | | **224 (33.3%)** | **149 (22.2%)** | **299 (44.5%)** |

Key: S = Sensitive R = Resistant I = Intermidate

DA = Clindamycine, AMP = Ampicillin; AMC = Amoxicillin-clavulanic acid;

GEN = Gentamicin; K = Kanamycin, CIP = Ciprofloxacin; CHL = Chloranphenicol; NA = Nalidixic acid ; CRO = Ceftriaxone ; F =Nitrofurantoin and S = Streptomycine, TE= Tetracycline

Figure S1: Antimicrobial susceptibility pattern of salmonella isolate at Wolaita Sodo Municipal Abattoir, 2016.
